# Conditional immobilization for live imaging *Caenorhabditis elegans* using auxin-dependent protein depletion

**DOI:** 10.1093/g3journal/jkab310

**Published:** 2021-09-02

**Authors:** Cori K Cahoon, Diana E Libuda

**Affiliations:** Department of Biology, Institute of Molecular Biology, University of Oregon, Eugene, OR 97403-1229, USA

**Keywords:** worms, live imaging, meiosis, spermatogenesis, oogenesis, germ cell development, gametogenesis, auxin-inducible degron system, germline, *C. elegans*

## Abstract

The visualization of biological processes using fluorescent proteins and dyes in living organisms has enabled numerous scientific discoveries. The nematode *Caenorhabditis elegans* is a widely used model organism for live imaging studies since the transparent nature of the worm enables imaging of nearly all tissues within a whole, intact animal. While current techniques are optimized to enable the immobilization of hermaphrodite worms for live imaging, many of these approaches fail to successfully restrain the smaller male worms. To enable live imaging of worms of both sexes, we developed a new genetic, conditional immobilization tool that uses the auxin-inducible degron (AID) system to immobilize both adult and larval hermaphrodite and male worms for live imaging. Based on chromosome location, mutant phenotype, and predicted germline consequence, we identified and AID-tagged three candidate genes (*unc-18*, *unc-104*, and *unc-52*). Strains with these AID-tagged genes were placed on auxin and tested for mobility and germline defects. Among the candidate genes, auxin-mediated depletion of UNC-18 caused significant immobilization of both hermaphrodite and male worms that was also partially reversible upon removal from auxin. Notably, we found that male worms require a higher concentration of auxin for a similar amount of immobilization as hermaphrodites, thereby suggesting a potential sex-specific difference in auxin absorption and/or processing. In both males and hermaphrodites, depletion of UNC-18 did not largely alter fertility, germline progression, nor meiotic recombination. Finally, we demonstrate that this new genetic tool can successfully immobilize both sexes enabling live imaging studies of sexually dimorphic features in *C. elegans*.

## Introduction

The discovery of green fluorescent protein (GFP) and subsequent proliferation of both engineered and additional fluorescent proteins has revolutionized biological research by enabling direct visualization of the biological processes occurring within living organisms. This breakthrough changed how scientists viewed cellular processes from snapshots in time generated by fixed images to the complete dynamic picture occurring in real time within the organism (Chalfie 2009). Live imaging experiments are now performed in nearly every model system spanning all kingdoms of life and can be found in a diverse range of biological fields from molecular biology to systems and synthetic biology (Remington 2011).

For decades, researchers have been curating protocols and technologies to facilitate imaging live *Caenorhabditis elegans* worms. This small, soil-dwelling nematode is completely transparent, thereby making it ideal for live imaging since the intact worm can be placed on a slide and imaged without requiring any tissue dissection or tissue clarification. However, immobilizing the worms on slides can be challenging since wild type worms are highly mobile and spend their life traveling in a sinusoidal pattern across agar plates eating lawns of bacteria. Further, worms display phototaxis when exposed to light stimulus, avoiding ultraviolet (UV; 340 nm), blue (470 nm), and green (500 nm) wavelengths of light (Ward *et al.* 2008). This phototaxis behavior is problematic for live imaging since most of these experiments visualize proteins with GFP, which is typically excited by 488-nm light. Thus, the stimulus used to excite GFP also triggers avoidance behavior from the worms.

To enable immobilization of *C. elegans*, custom microfluidic devices have been successfully used to keep worms immobile and alive for imaging experiments anywhere from hours to days (San-Miguel and Lu 2013). While these devices work very well at gently immobilizing the worms, the generation of these devices requires a fabrication facility with the appropriate equipment and expertise in photolithography, which is used to generate the negative molds that create the plastic microfluidic chips. Thus, for many labs without access to in-house fabrication facilities, the generation of microfluidic chips requires external outsourcing, which can be a slow and expensive process if multiple edits of the design are necessary.

Temperature-sensitive hydrogels have also been used effectively to immobilize worms for long timelapse imaging experiments. These hydrogels work by remaining in a viscous liquid state at cold temperature (typically around 4°C) and then solidify at room temperature. This method allows worms to be embedded into the cold hydrogel liquid and become immobilized as the gel warms (Dong *et al.* 2018). Additionally, worms stop thrashing upon cold exposure via ice or cooled liquids (*e.g.*, a cold hydrogel), which allows for manipulation and placement of the worm within the hydrogel (Chung *et al.* 2008). However, both ice and cold hydrogels can trigger acute cold shock, which is known to trigger a stress response that has negative consequences in worms, including morphological changes within the germline and gut (Robinson and Powell 2016). Therefore, this method is not an option if the worms need to be exposed to continuous elevated temperatures either to maintain the mutant phenotype or to study the heat shock stress response pathway.

UV crosslinked hydrogels circumvent the need for temperature changes to solidify the gel. Upon exposure to a UV lamp, UV-sensitive hydrogels crosslink together and immobilize worms for long timelapse imaging (Burnett *et al.* 2018; Han *et al.* 2021). While this method will not generate any temperature stress responses, exposure to UV causes DNA damage, which can lead to multiple downstream cellular stress events including apoptosis (Mullenders 2018). Thus, UV-sensitive hydrogels may not be an ideal option for studying particular biological processes, such as the maintenance of genome integrity.

The use of agar pads, anesthetics, and polystyrene beads are widely used to immobilize worms. Also, the small size of *C. elegans* adult hermaphrodite, ∼1 mm long and ∼80 µm wide, is about the size of the groove in a vinyl record and imprinting these grooves onto an agar pad is effective at immobilizing hermaphrodites when used in combination with anesthetics and polystyrene beads (Rivera Gomez and Schvarzstein 2018). Anesthetics, such as acetylcholine receptor antagonists, have the potential to suppress and/or slow the pharyngeal pumping of the worm, which is analogous to the pumping of the mammalian heart. If pharyngeal pumping is suppressed for extended periods of time, then the worm will die. Thus, depending on the specific live imaging experiment, the addition of anesthetics may not be an option. Further, the polystyrene beads, which are usually around 0.1 µm and prevent squashing the worm between the coverslip and the agar pad, are small enough for the worm to ingest (Avery and Shtonda 2003; Nika *et al.* 2016). Currently, it is unclear what the consequences are for the health of a worm when it ingests polystyrene and how this ingestion might affect some biological processes.

In comparison to hermaphrodites, *C. elegans* males are both smaller in length (∼0.8 mm) and width (∼50 µm). Consequently, many methods that immobilize the hermaphrodites fail to immobilize males. *Caenorhabditis* *elegans* is an excellent model system for sexual dimorphism studies with many sex-specific differences having been identified related to germline processes, nervous system development, and animal aging (Chu *et al.* 2006; Stanfield and Villeneuve 2006; Jaramillo-Lambert *et al.* 2007; Shakes *et al.* 2009; Jaramillo-Lambert *et al.* 2010; Checchi *et al.* 2014; Jaramillo-Lambert *et al.* 2016; Barr *et al.* 2018; Hotzi *et al.* 2018; Cahoon and Libuda 2019; Kurhanewicz *et al.* 2020; Li *et al.* 2020). Due to the differences in body sizes between sexes, it has been difficult to do live imaging-based experiments to analyze and compare the sexually dimorphic features within *C. elegans*. Thus, we developed a genetic immobilization tool that works for both male and hermaphrodite worms. Using the auxin-inducible degron (AID) system, we designed a conditional immobilization system where worms are only paralyzed upon exposure to the plant hormone auxin. Here, we validate this conditional immobilization tool using the *C. elegans* germline and demonstrate that this system works efficiently in both sexes. Notably, we also found that male worms require a higher concentration of auxin to display the same amount of paralysis as hermaphrodites, thereby revealing a potential novel sexual dimorphism within the AID system.

## Methods

### *Caenorhabditis elegans* strains, genetics, CRISPR, and culture conditions

All strains were generated from the N2 background and were maintained and crossed at 20**°**C under standard conditions on nematode growth media (NGM) with lawns of *Escherichia coli*. InVivo Biosystems tagged *unc-104*, *unc-18*, and *unc-52* with the AID [AID*, asterisk denotes the 44 amino acid tag rather than the 68 amino acid miniAID (mAID) tag (Ashley *et al.* 2021)] tag (amino acid sequence: PKDPAKPPAKAQVVGWPPVRSYRKNVMVSCKSSGGPEAQAAFVK) using CRISPR/Cas9. Both *unc-104* and *unc-18* were tagged on the C-terminus and *unc-52* was tagged on the N-terminus. For all genes, the CRISPR homology-directed repair template was constructed containing 35 base pairs of homology on either side of the insertion site with a small recoded section at the sgRNA site to avoid Cas9 cutting the template. Additionally, a GSTGS amino acid linker was included between the AID* tag and each gene. These repair constructs were synthesized as oligos and injected into the worm with two sgRNAs for each gene. All sequences and screening primers for the CRISPR/Cas9 tagging of these genes are in Supplementary Table S1. All CRISPR/Cas9 worm lines were backcrossed to N2 worms three times before processing with any strain construction.

The following strains were used in this study:

**Table:** 

N2:	Bristol wild type strain.
COP2270:	unc-52(knu968[AID*::unc-52]) II.
COP2271:	unc-18(knu969[unc-18::AID*]) X.
COP2281:	unc-104(knu973 [unc-104::AID*]) II.
CV586:	*syp-2::GFP IV.*
DLW109:	*lib23*[eft-3p::TIR1::F2A::mTagBFP2::NLS::AID*::tbb-2 3’UTR::loxP] I. unc-104 (knu973 [unc-104::AID*]) oxSi1091[Pmex-5::Cas9(smu-2 introns) unc-119+]/unc-104 (knu973 [unc-104::AID*]) II.
DLW112:	reSi7[rgef-1p::TIR1::F2A::mTagBFP2::NLS::AID*::tbb-2 3’UTR] I. unc-104 (knu973 [unc-104::AID*]) II.
DLW114:	unc-18(knu969[unc-18::AID*]) X. reSi7 [rgef-1p::TIR1::F2A::mTagBFP2::NLS::AID*::tbb-2 3’UTR] I.
DLW118:	unc-18(knu969[unc-18::AID*]) X. reSi7 [rgef- 1p::TIR1::F2A::mTagBFP2::NLS::AID*::tbb-2 3’UTR] I. syp-2::GFP IV.
DLW124:	wrdSi22[eft-3p::TIR1::F2A::mTagBFP2::NLS::AID*::tbb-2 3’UTR::SEC[loxP; let-858term; sqt-1(d); hs::Cre; hygR; unc-54term; loxP]] I. unc-52(knu968[AID*::unc-52]) oxSi1091[Pmex-5::Cas9(smu-2 introns) unc-119+] II.
DLW148:	unc-18(knu969[unc-18::AID*]) X. reSi7 [rgef-1p::TIR1::F2A::mTagBFP2::NLS::AID*::tbb-2 3’UTR] I. oxSi487[mex-5p::mCherry::H2B::tbb-2: 3'UTR::gpd-2 operon::GFP::H2B::cye-1 3'UTR + unc-119(+)] II.
DV3805:	reSi7[rgef-1p::TIR1::F2A::mTagBFP2::NLS::AID*::tbb-2 3’UTR] I.
CB4108:	*fog-2(q71) V.*
JDW118:	*wrdSi22*[eft-3p::TIR1::F2A::mTagBFP2::NLS::AID*::tbb-2 3’UTR::SEC[loxP; let-858term; sqrt-1(d); hs::Cre; hygR; unc-54term; loxP]] I. oxSi1091 [Pmex-5::Cas9(smu-2 introns) unc-119+] II. unc-119(ed3) III.

### Worm tracking

L4 progeny from parental worms on either NGM or NGM with auxin [1 or 10 mM auxin (Naphthaleneacetic Acid (K-NAA), PhytoTechnology Laboratories, cat no. N610) (Martinez *et al.* 2020)] were transferred to new plates and imaged 18–24 h later as adults. Worms were imaged using a 1.3-megapixel eyepiece DinoCam camera on a Leica stereoscope with 0.75X magnification. Each movie was captured for 5–10 min at 15 frames per second. Using FIJI, movies were background subtracted with a rolling ball radius of 15.0 pixels with a sliding paraboloid and no smoothing. Then, a binary image mask of the worms was generated to enable tracking using the FIJI plugin wrMTrck (Schindelin *et al.* 2012; Nussbaum-Krammer *et al.* 2015). Only worms with long, consecutive durations of tracking were included in the analysis with worms that were tracked for less than 100 frames being excluded from the dataset. Average speed was determined by the length of the worm traveled in pixels distance divided by the total time the worm was tracked in seconds.

### Fertility assay

To assess fertility, L4 hermaphrodite worms grown on auxin were placed onto new 1 mM auxin plates and were transferred every 24 h for a total of 5 days. After 3 days from removing the hermaphrodite, each plate was scored for living progeny, dead eggs, and unfertilized eggs. For each genotype, 12 hermaphrodites were assayed for fertility. If a hermaphrodite went missing, died, or bagged during any of the 5 days, then all of her progeny count data were excluded from the dataset. Brood size was calculated for each genotype by summing the living progeny with the dead eggs.

### Male mating assay

Males were grown on auxin as described below in “Conditional immobilization for live imaging.” Immobilized L4 males were picked to new 10 mM auxin plates and 24 h later, young adult males were removed from auxin and mated to young adult *fog-2* females, which are obligate females and lack a spermatheca (Schedl and Kimble 1988). Since *fog-2* young adult females do not lay fertilized embryos unless a successful mating has occurred, mating efficiency of the recovered unc-18::AID* males was determined by the presence of embryos on the plates at 24 h postmating.

### Conditional immobilization for live imaging

For live imaging experiments, parental worms were placed on plates containing NGM + 1 mM auxin or 10 mM auxin. For all male experiments, the parental worms were first picked as L4s onto NGM plates and left to mate for 18–24 h prior to moving the worms to 10 mM auxin NGM plates. From the mated parental worms, L4 progeny grown on auxin were transferred to new auxin plates and young adult progeny worms were used for all imaging experiments. (Note: shorter exposures of auxin, such as 24 h, did result in some minor paralysis for unc-18:AID* strains but this phenotype was not as strong of a paralysis phenotype as growth on auxin for a full generation) L4 worms from SYP-2::GFP strains were placed at 25°C overnight to enhance GFP expression (Rog and Dernburg 2015; Pattabiraman *et al.* 2017). For mounting of live worms, five to six worms were picked onto poly-lysine coated coverslips [Sigma-Aldrich, cat no. P8920; performed as described in Wang *et al.* (2018)] (Supplementary Figure S1). Worms were placed into 1–2 µL of imaging media containing M9 media with 25 mM serotonin [Sigma-Aldrich, cat no. H7752 (Rog and Dernburg 2015)] and either 1 mM auxin (hermaphrodites) or 10 mM auxin (males). To prevent the worms from floating in the imaging media, a 7–9% agarose (Invitrogen, cat no. 16500500) pad made from the imaging media was gently placed over the top of the worms. Agarose pads were generated placing a drop of the melted agarose between two coverslips. Then, the solidified pad was lifted with a spatula and gently laid down over the worms. A piece of torn Whatman paper was used to wick away excess liquid and prevent the agarose pad from floating. A ring of Vaseline was placed around the agarose pad to attach the microscope slide that was placed on top of the coverslip-worm-agarose pad sandwich, and to prevent drying out of the pad while imaging. Then, worms were imaged (see microscopy section). Postimaging, the worms were monitored for recovery by gently removing the slide from the Vaseline seal with a needle or spatula and either transferring the agarose pad with the worms into a glass well dish filled with M9 media or inverting the pad to pipette M9 media directly on top of the worms. Then, worms were carefully pipetted with low bind tips (Genesee Scientific, cat no. 23-121RS) to NGM plates. Worms were monitored for recovery at 24 h postimaging. Any worms that did not survive after the recovery were excluded from any analysis. For experiments with worms imaged at 60X, 0.08% tricaine (Ethyl 3-aminobenzoate methanesulfonate; Sigma-Aldrich, cat. no. E10521-50G) and 0.008% tetramisole hydrochloride (Sigma-Aldrich, cat. no. T1512-10G) were added to the M9 imaging media and agarose pads. The addition of the anesthetics did not interfere with the recovery of the worms postimaging.

### Immunohistochemisty

Immunofluorescence was performed as described in Libuda *et al.* (2013). Briefly, gonads were dissected in egg buffer with 0.1% Tween20 on to VWR Superfrost Plus slides from 18 to 24 h post-L4 worms on NGM only plates or NGM plates with 1 or 10 mM auxin (see “Conditional immobilization for live imaging” for details on how worms were grown on auxin). Dissected gonads were fixed in 5% paraformaldehyde for 5 min, flash-frozen in liquid nitrogen, and fixed for 1 min in 100% methanol at −20°C. Slides were washed three times in PBS + 0.1% Tween20 (PBST) for 5 min each and incubated in block (0.7% bovine serum albumin in PBST) for 1 h. Primary antibodies [rabbit anti-RAD-51, 1:1500 (Kurhanewicz *et al.* 2020; Toraason *et al.* 2021)] were added and incubated overnight in a humid chamber with a parafilm cover. Slides were then washed three times in PBST for 10 min each and incubated with secondary antibodies (goat anti-rabbit AlexaFluor488, ThermoFisher, cat. no. A11034) at 1:200 dilution for 2 h in a humid chamber with a parafilm cover. Slides were washed two times in PBST then incubated with 2 µg/mL DAPI for 15–20 min in a humid chamber. Prior to mounting slides were washed once more in PBST for 10 min and mounted using Vectashield with a 22 × 22 mm coverslip (no. 1.5). Slides were sealed with nail polish and stored at 4°C until imaged.

EdU staining was performed as described in Almanzar *et al.* (2021) with minor changes. Briefly, worms were washed three times in PBS + 0.1% TritonX. Then, worms were incubated for 1.5 h nutating in PBS + 0.1% TritonX with 4 mM 5-Ethynyl-2’-deoxyuridine (EdU), which was diluted from a stock 10 mM EdU in distilled water from the Invitrogen Click-iT Edu Alexa Fluor 488 imaging kit (Invitrogen, cat. no. C10338). Worms were washed two times in PBS + 0.1% TritonX for 1–2 min each then plated onto either NGM or NGM with 1- or 10-mM auxin plates. Time was noted when worms were removed from EdU to start chase time course of the EdU staining. Both male and hermaphrodite worms were dissected and fixed as described above at 0, 10, and 24 h postremoval from EdU and only hermaphrodites were dissected at 48 h post-EdU removal. At each time point, 15–20 worms were dissected and washed three times in PBST. Then, slides were either immediately processed with the Click-iT reaction or held in PBST overnight at 4°C and the Click-iT reaction was performed the next day. The Click-iT reaction was performed as described in the kit manual except the volumes in the Click-iT reaction mix were reduced. All slides were incubated with 50 µL of the Click-iT reaction mix containing 43 µL 1X Click-iT reaction buffer, 2 µL CuSO_4_, 0.2 µL AlexaFluor488, and 5 µL reaction buffer additive. Slides were incubated for 30 min in a humid chamber with a parafilm cover. Then washed three times in PBST for 10 min each and incubated with 2 µg/mL DAPI in water for 20 min with a parafilm cover. Slides were washed once in PBST for 10 min then mounted in Vectashield using 22 × 22 mm coverslip (no. 1.5) and sealed using nail polish. All slides were stored at 4°C and imaged within 1–2 days.

### Microscopy

Immunofluorescence slides of gonad stained with RAD-51 were imaged on a GE DeltaVision microscope with a 63X/N.A. 1.42 lens and 1.5X optivar at 1024 × 1024 pixel dimensions. Images were acquired using 0.2 µm Z-step size and deconvolved with softWoRx deconvolution software. EdU slides and all brightfield timelapses were imaged on GE IN Cell microscope. Edu slides were imaged with a 40X/N.A. 0.95 lens using a Z-step size of 0.72 µm, male brightfield timelapses were imaged with 20X/N.A. 0.75 lens using a Z-step size of 1.3 µm, and hermaphrodite brightfield timelapses were imaged 10X/N.A. 0.45 lens using a Z-step size of 3.91 µm. All IN Cell timelapses and images were deconvolved using the IN Cell 3D deconvolution software. SYP-2::GFP, L1 autofluorescence, and mCherry::H2B timelapses were imaged on a Nikon CSU SoRa Spinning Disk Microscope with a 60X water lens/N.A. 1.2 using a Z-step size of 0.3 µm.

### Image analysis and quantification

RAD-51 gonad images were stitched together using the FIJI (NIH) plugin Stitcher (Preibisch *et al.* 2009) and analyzed in Imaris (Oxford Instruments) as described in Toraason *et al.* (2021) with minor changes. Each gonad from the transition zone through the end of pachytene was analyzed for RAD-51 foci per nucleus, which was determined by DAPI morphology. The start of the transition zone and pachytene was defined by the first row that did not contain more the 1–2 nuclei of either premeiotic nuclei (compact nuclei) or transition zone nuclei (nuclei with DNA in a polarized or “crescent” shape morphology), respectively. The end of pachytene was defined by the last row that contained all pachytene nuclei with the occasional single diplotene nucleus. The pachytene region was then equally divided into three zones based the length of this region within the germline to generate early pachytene, mid pachytene, and late pachytene. These criteria were used for establishing the transition zone, early pachytene, mid pachytene, and late pachytene in both hermaphrodites and males. To determine the position of the EdU staining within the germline, the EdU gonad images were max intensity *z*-projected in FIJI. Then, the position of the EdU straining front was determined by the last nucleus within the germline labeled with EdU. Max intensity *z*-projection montages and movies were made in FIJI, and only GFP::SYP-2 movies were stabilized using the FIJI plugin “StackRegJ” (https://research.stowers.org/imagejplugins/ ; last accessed 9/2021). This stabilization was necessary to reduce the motion of the germline inside the worm and generate a stable movie for viewing. Additionally, photobleach correction was applied to the GFP::SYP-2 male movie using the photobleach correction application in FIJI. All images and movies have been slightly adjusted for brightness and contrast using FIJI.

### Statistics

All statistical tests were performed using Prism. For the worm tracking assay, the average speed of each worm was calculated in the FIJI plugin “wrMTrck” and the multiple comparisons Kruskal–Wallis/Dunn’s multiple comparisons test was performed to determine statistical differences between each genotype assayed. For the fertility assay, brood size was determined by summing living progeny and dead eggs and statistical differences were determined using two-way ANOVA with Dunnett’s multiple comparisons test. For RAD-51, statistical differences were determined the Kruskal–Wallis/Dunn’s multiple comparisons nonparametric ANOVA test was used. For DAPI body quantification, statistical differences were determined using the nonparametric Mann–Whitney test. Each test used is indicated in the *Results* section next to the reported *P*-value and all *n* values are reported in the figure legends.

## Results

### Reversible paralysis from auxin-dependent depletion of UNC-18 and UNC-104

**Figure 1:**
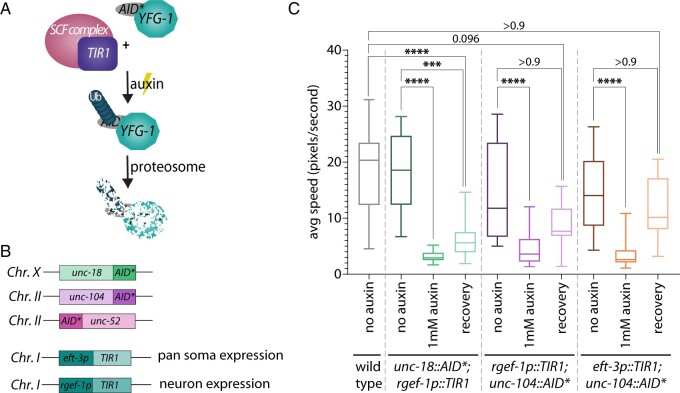
Auxin-dependent depletion of *unc-18* and *unc-104* permits conditional immobilization of hermaphrodites. (A) Diagram of the AID system showing how *TIR1* associates with the endogenous SCF E3 ligase complex that in the presence of auxin cause ubiquination (Ub) of the AID*::YFG-1 protein (Your Favorite Gene 1). This ubiquination results in proteosomal degradation of AID*::YFG-1. (B) Schematics of the three candidate genes CRISPR/Cas9 tagged with AID* and the *TIR1* constructs used with the expression location of *TIR1* based on the *eft-3* or *rgef-1* promoters. The chromosome number where each construct is located in the genome is indicated on the left of each schematic. (C) Quantification of the average speed in pixels per second of wild type (*n* = 30), unc-18::AID*; rgef-1p::TIR1 (no auxin *n* = 18, 1 mM auxin *n* = 16, recovery *n* = 26), rgef-1p::TIR1; unc-104::AID* (no auxin *n* = 24, 1 mM auxin *n* = 31, recovery *n* = 18), and eft-3p::TIR1; unc-104::AID* (no auxin *n* = 15, 1 mM auxin *n* = 26, recovery *n* = 24) on plates containing no auxin and 1 mM auxin. The recovery category indicates worms that have been off auxin for 18–24 h prior to tracking worm motion. **** indicates *P* < 0.00001 and *** indicates *P* < 0.0001, Kruskal–Wallis/Dunn’s multiple comparisons.

To conditionally immobilize worms, we used the AID system, which has been used in multiple different worm tissues to selectively deplete proteins of interests at specific stages of development (Zhang *et al.* 2015; Pelisch *et al.* 2017; Kasimatis *et al.* 2018; Serrano-Saiz *et al.* 2018; Martinez *et al.* 2020; Ashley *et al.* 2021). This system requires three components: (1) a protein containing the AID sequence; (2) expression of the plant F box protein Transport Inhibitor Response 1 (TIR1), which can be regulated using tissue-specific promoters; and (3) the plant hormone auxin, which can be absorbed externally by the worms (Figure 1A). Auxin exposure promotes the binding of TIR1 to the degron sequence. TIR1 is able to interact with components of the endogenous SCF E3 ubiquitin ligase complex generating a functional complex that can ubiquitinate the degron tagged protein and target it for proteasome-mediated degradation (Natsume and Kanemaki 2017). Additionally, this protein degradation is completely reversible once the worms are removed from auxin, such that after a period of time the degron tagged protein can return to wild type levels if mRNA transcripts are present (Zhang *et al.* 2015).

To conditionally immobilize worms, we combined the AID system with genes that cause severe worm paralysis when mutated and applied this immobilization to visualize the germline in live animals. To narrow down the candidate list of genes, we focused on Chromosomes *X* and *II* since we wanted to use this system to study the *C. elegans* germline and these chromosomes are mainly devoid of germline expressed genes (Reinke *et al.* 2000). We then obtained mutants from all the identified genes on these chromosomes that were indicated at the CGC as being homozygous viable and severely paralyzed. Additionally, we avoided any genes that had the potential to alter the germline, vulval development, or vulval function. From these candidates, we selected three genes to tag with the AID sequence: *unc-104*, *unc-18*, and *unc-52* (Figure 1B). UNC-104 is a kinesin-3 family motor protein that is primarily involved in transporting synaptic vesicle precursors within neurons (Hayashi *et al.* 2019). UNC-18 belongs to the Sec1p/Munc18 family proteins and plays a critical role in synaptic exocytosis in neurons (Park *et al.* 2017). UNC-52 is an extracellular matrix heparan sulfate proteoglycan that plays an essential role in myofilament assembly of body-wall muscles (Rogalski *et al.* 2001).

Using CRISPR/Cas9, each of these genes was tagged with the AID sequence and genetic crosses were performed to incorporate *TIR1*. We used the 44 amino acid version of the AID tag for tagging all three genes, which we denote on all our strains as AID* to differentiate from the 68 amino acid mAID tag (Ashley *et al.* 2021). Two different *TIR1* constructs were used in this study: (1) *rgef-1p::TIR1*, which expresses only in neurons; and (2) *eft-3p::TIR1*, which has pan-somatic expression. For all experiments, we found that animals needed to be grown for a single generation on plates containing NGM with auxin to exhibit the strongest paralysis phenotype (see *Methods*).

We first assayed unc-104::AID* and unc-18: AID* strains using the neuron-specific expression of *TIR1 (rgef-1p::TIR1)* and AID*::unc-52 with pan-somatic expression of *TIR1 (eft-3p::TIR1)* (Figure 1B). AID*::unc-52 displayed no changes in mobility when grown on auxin plates, thus this gene was excluded from any further studies. Both unc-104::AID* and unc-18::AID* strains display significant decreases in mobility on auxin plates, which we assayed by tracking the motion of the worms on normal NGM plates and NGM with 1 mM auxin (Figure 1C, *P* < 0.0001, Kruskal–Wallis/Dunn’s multiple comparisons). We noticed that unc-104::AID* did not display as strong of a mobility defect as unc-18::AID* when depleted using the neuron-specific *TIR1* (median average speed: 3.604 and 2.927 pixels/s, respectively). Moreover, depleting UNC-104::AID* with the pan-somatic *eft-3* driven TIR1 exhibited a similar degree of mobility defects to neuron-specific depletion of UNC-104::AID* with the *rgef-1* driven TIR1 (*P* > 0.999, Kruskal–Wallis/Dunn’s multiple comparisons). Although the effects of each driver on UNC-104::AID* depletion were statistically indistinguishable, the median average speed was slightly lower with the pan-somatic *eft-3* driver (neuron-specific *rgef-1* driven TIR1: 3.604 pixels/s; pan-somatic *eft-3* driven TIR1: 2.625 pixels/s).

With this worm immobilization technique, worms can be recovered postimaging and assayed for viability off auxin. We found that upon removal from auxin for 18–24 h both unc-104::AID* and unc-18::AID* worms recovered some degree of normal motion (Figure 1C). unc-104::AID* with *TIR1* driven by both the neuron-specific *rgef-1* promoter or the pan-somatic *eft-3* promoter recovered motion to levels statistically indistinguishable from wild type and the no auxin cohort (wild type compared to neuronal-specific *P* > 0.9 and pan-somatic *P* = 0.096; no auxin control compared to neuronal-specific *P* > 0.9 and pan-somatic *P* > 0.9, Kruskal–Wallis/Dunn’s multiple comparisons). However, the overall median average speed in recovered unc-104::AID* animals was lower than wild type worms (wild type: 20.34 pixel/s; rgef-1p:TIR1; unc-104::AID*: 7.669 pixel/s; eft-3p::TIR1; unc-104::AID*: 10.16 pixel/s). unc-18::AID* worms were able to recover some motion off auxin compared to the unc-18::AID* in the presence of auxin; however, this recovered motion was significantly slower than wild type worms (median average speed for unc-18::AID* on auxin: 2.927 pixel/s; unc-18::AID* recovery: 5.634 pixel/s; wild type: 20.34 pixel/s; *P* < 0.0001, Kruskal–Wallis/Dunn’s multiple comparisons) and the no auxin cohort (median average speed = 18.58 pixel/s; *P* = 0.0002, Kruskal–Wallis/Dunn’s multiple comparisons). Taken together, these motion recovery experiments suggest that unc-18::AID* may require more time to completely recover wild type motion compared to the unc-104::AID* worms. Overall, both unc-104::AID* and unc-18::AID* are able to both partially recover movement after removal from the auxin treatment.

### UNC-104::AID* depletion has slight behavioral and fertility defects

**Figure 2:**
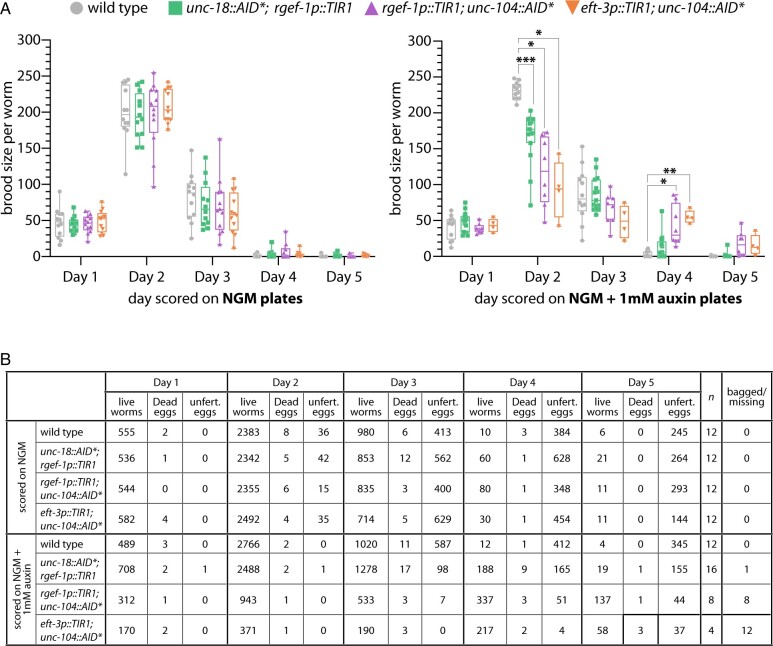
Auxin-dependent depletion of UNC-18 does not significantly alter fertility. (A) Brood size calculations and (B) progeny counts of lives worms, dead eggs, and unfertilized (unfert.) eggs for wild type, unc-18::AID*; rgef-1p::TIR1, rgef-1p::TIR1; unc-104::AID*, and eft-3p::TIR1; unc-104::AID* on NGM with and without 1 mM auxin. *N*-value indicates the number of parental hermaphrodites scored and bagged/missing indicates the number of worms that bagged or disappeared from the plate during the 5 days of scoring. * indicates *P* < 0.01, ** indicates *P* < 0.001, *** indicates *P* < 0.0001, Dunnett’s multiple comparisons test.

To examine the effectiveness of this conditional immobilization system for live imaging, we focused on implementing this system for live imaging the *C. elegans* germline. Since the effects of UNC-104 or UNC-18 loss on germline function are unknown, we examined multiple aspects of germline biology to determine if a loss of UNC-104 or UNC-18 causes germline-specific defects. We began by assaying the fertility of hermaphrodite worms containing unc-104::AID* and unc-18::AID* under both depletion (in the presence of auxin) and wild type conditions (Figure 2). For these fertility assays, we counted the number of living progeny, dead eggs, and unfertilized eggs from hermaphrodite worms that were moved each day for 5 days to new plates (see *Methods*). Scoring fertility over multiple days allowed for observation of most of the hermaphrodite reproductive lifespan, which begins with a large abundance of living progeny and subsequently ends with unfertilized eggs once the hermaphrodite sperm is depleted (Ward and Carrel 1979). To compare the fertility of each genotype, we calculated brood size of each worm, which is the sum of living progeny and dead eggs.

In the absence of auxin, all genotypes displayed brood sizes that were indistinguishable from wild type worms through all 5 days of scoring (Figure 2A). In particular, wild type worms display similar brood sizes both on and off auxin throughout all 5 days. Additionally, the number of dead eggs was also similar between the on and off auxin wild type worms suggesting that oocyte viability is not being altered (Figure 2B). Further, the cumulative sum of the brood size for wild type both on and off auxin (no auxin average cumulative brood size for 12 worms: 329.417 ± 62.2 SD; auxin average cumulative brood size for 12 worms: 360.917 ± 25.6 SD) is comparable to published results showing that wild type animals have a cumulative total brood size of ∼300 worms (Ward and Carrel 1979; Muschiol *et al.* 2009). Moreover, previous studies have found as well that auxin exposure does not alter the viability or fertility of wild type worms (Zhang *et al.* 2015; Martinez *et al.* 2020; Ashley *et al.* 2021). Taken together, this mounting evidence supports a conclusion the auxin does not affect the fertility or germline progression in wild type worms.

In the presence of auxin, unc-104::AID* worms displayed slight changes in fertility (Figure 2A). The progeny laid on auxin plates during the first 24 h (Day 1) displayed no significant changes in brood size for any of the genotypes examined compared to wild type animals. Over the next 24 h (Day 2) on auxin, unc-104::AID* displayed significant reductions in brood size compared to wild type (rgef-1p::TIR1; unc-104::AID* *P* = 0.0135; eft-3p::TIR1; unc-104::AID* *P* = 0.0158, Dunnett’s multiple comparisons). In contrast, on days four and five unc-104::AID* displayed larger brood sizes than wild type animals with these changes being only significantly different on Day 4 (Day 4: rgef-1p::TIR1; unc-104::AID* *P* = 0.0447; eft-3p::TIR1; unc-104::AID* *P* = 0.0036, Dunnett’s multiple comparisons; Day 5: rgef-1p::TIR1; unc-104::AID* *P* = 0.1344; eft-3p::TIR1; unc-104::AID* *P* = 0.1944, Dunnett’s multiple comparisons). This result suggests that depletion of UNC-104 might cause a slight delay in oocyte progression through the germline allowing more oocytes to be laid later during the hermaphrodite reproductive lifespan. Further experiments are necessary to determine if indeed this is a delay or if something else is contributing to the discrepancies in brood size compared to wild type animals.

In addition to having significant changes in brood size on auxin, unc-104::AID* worms also displayed a high number of worms with a bag of worms phenotype, where worms retained their progeny within their body cavity, and worms that went missing over the 5 days of scoring (Figure 2B). Additionally, unc-104::AID* worms on auxin displayed behavioral defects where instead of staying within the bacteria lawn on the plate these worms exhibited a wandering pattern outside of the bacterial lawn that was abnormal for hermaphrodites. Typically, hermaphrodite worms remain within the bacteria lawn and rarely wander to the edges of the plate (Lipton *et al.* 2004). However, the unc-104::AID* worms on auxin would drag themselves in nonsinusoidal patterns to the edges of the plates where they would stick to the plastic and desiccate before dying. Taken together, these data suggest that the unc-104::AID* worms may have both a behavioral defect and a slight fertility defect or delay. Due to these defects, we decided to exclude unc-104::AID* for use in any future live imaging experiments and proceeded with the analysis of unc-18::AID* worms.

### UNC-18::AID* depletion has minimal effects in the hermaphrodite germline

In the presence of auxin, unc-18::AID* worms displayed slight changes in brood size, but the brood viability (dead eggs) remains indistinguishable from wild type (Figure 2). The progeny laid from unc-18::AID* worms during the first 24 h (Day 1) on auxin exhibited no significant changes in brood size. In contrast, the progeny on auxin at Day 2 displayed significant reductions in brood size compared to wild type (unc-18::AID*; rgef-1p::TIR1 *P* = 0.0002, Dunnett’s multiple comparisons). Notably, this decrease in brood size on Day 2 did not correlate with an increase in the number of dead eggs, indicating that oocyte viability is not the likely cause of this brood size reduction (Figure 2B). Further, over the next 3 days of scoring, the brood size of unc-18::AID* worms was indistinguishable from wild type worms on auxin suggesting that this slight reduction only effects the 24–48 h progeny. Additionally, unc-18::AID* worms displayed none of the behavioral or worm bagging defects that were seen with unc-104::AID* animals (Figure 2B).

**Figure 3:**
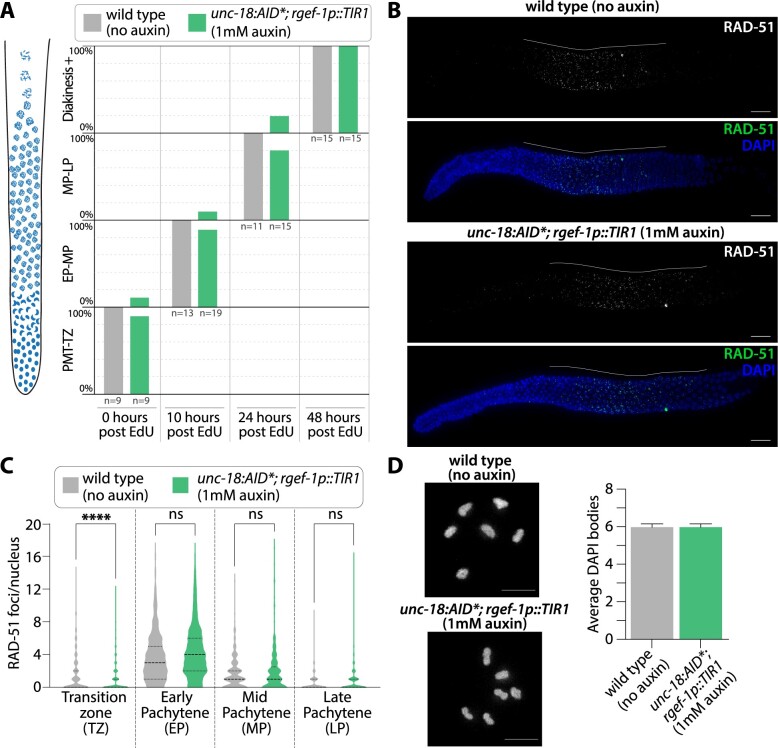
Hermaphrodite germline progression and meiotic crossover formation are unaffected by auxin-depletion of *unc-18.* (A) Quantification of the nuclear progression through the germline in wild type with no auxin exposure (grey) and unc-18::AID*; rgef-1p::TIR1 on 1 mM auxin plates (green). Each gonad was scored based on the germline position of the last EdU stained nucleus from gonads dissected at 0, 10, 24, and 48 h post-EdU labeling. For this analysis, the germline (diagrammed on the left) was divided into four regions: premeiotic tip to transition zone (PMT-TZ), early pachytene to mid pachytene (EP-MP), mid pachytene to late pachytene (MP-LP), and diakinesis to end of the germline (Diakinesis+). *N*-values for the number of worms scored are displayed on the plot below each bar. (B) Representative images of dissected gonads from wild type (no auxin exposure) and unc-18::AID*; rgef-1p::TIR1 (1 mM auxin exposure) hermaphrodites stained for RAD-51 (green) and DNA (DAPI, blue). Scale bar represents 20 µm and white line indicates the length of the RAD-51 staining within the germline. (C) Quantification of the number of RAD-51 foci per nucleus for wild type (grey, no auxin exposure, *n* = 6 gonads, 791 TZ nuclei, 352 EP nuclei, 284 MP nuclei, 253 LP nuclei) and unc-18::AID*; rgef-1p::TIR1 (green, 1 mM auxin exposure, *n* = 6 gonads, 615 TZ nuclei, 422 EP nuclei, 261 MP nuclei, 224 LP nuclei). Statistics determined using Kruskal–Wallis/Dunn’s multiple comparisons with * representing the number of significant digits after the decimal and “ns” meaning not significant. (D) Representative image and quantification of diakinesis chromosomes (DAPI staining bodies) from wild type (no auxin exposure, *n* = 30 oocytes) and unc-18::AID*; rgef-1p::TIR1 (1 mM auxin exposure, *n* = 30 oocytes).

The slight reduction in the 24–48 h brood when UNC-18 is depleted could be explained by changes in germline progression. To assess for changes in germline progression, we used an EdU pulse-chase experiment where worms were soaked in EdU (“pulse”) to allow EdU incorporation into nuclei undergoing DNA replication, including those in the premeiotic tip. Then, worms were dissected at 0, 10, 24, and 48 h post-EdU soaking to “chase” the EdU staining in the germline (see *Methods*) (Crittenden *et al.* 2006; Jaramillo-Lambert *et al.* 2007; Morgan *et al.* 2010; Fox *et al.* 2011; Rosu *et al.* 2011; Seidel and Kimble 2015; Almanzar *et al.* 2021). To score EdU progression, the *C. elegans* germline was divided up into four different regions based on nuclear DNA morphology: premeiotic to transition zone (PMT-TZ), early pachytene to mid pachytene (EP-MP), mid pachytene to late pachytene (MP-LP), and diakinesis to germline end (Diakinesis+) (Hillers *et al.* 2017). Since the results of our fertility assays (Figure 2) and those of multiple studies have found that auxin has no effect on the viability and/or fertility in wild type worms (Zhang *et al.* 2015; Martinez *et al.* 2020; Ashley *et al.* 2021), we performed all the EdU experiments comparing wild type worms in the absence of auxin to unc-18::AID* worms in presence of auxin, unless otherwise indicated. For our EdU pulse-chase experiments, both wild type and unc-18::AID* displayed very similar patterns of EdU staining throughout the time course with nuclei appearing to progress at similar rates (Figure 3A). Germlines initially show staining in the PMT-TZ region, which is indicative of the mitotic and meiosis S phase DNA replication occurring in this region. Then, the EdU front slowly progresses through each germline stage from EP-MP at 10 h, MP-LP at 24 h, and past diakinesis (Diakinesis+) at 48 h. Overall, the progression of nuclei through the germline is not grossly altered by auxin-mediated depletion of UNC-18::AID*.

To determine if meiotic recombination is altered by depletion of UNC-18::AID*, we assayed for the initiation of recombination through the quantification of meiotic double-strand DNA breaks (DSBs) using the recombinase protein RAD-51. At the onset of meiosis, the topoisomerase-like enzyme SPO-11 induces DSBs from which a subset are repaired via homologous recombination into crossover events, which ensure accurate segregation of the homologs during meiosis I (reviewed in Hunter 2015). In *C. elegans*, DSBs are formed during the transition zone through early pachytene, and then RAD-51 is loaded on to the resected single-stranded DNA ends of the DSB site (Hillers *et al.* 2017). As the nuclei progress through pachytene, the DSB is repaired by homologous recombination and RAD-51 is removed from the DSB site (Hillers *et al.* 2017). Using immunofluorescence, we quantified the amount of RAD-51 foci within the germline from the beginning of the transition zone to the end of pachytene in both wild type and unc-18::AID* worms (Figure 3, B and C). Nuclear morphology based on DAPI staining was used to define each region and pachytene was equally divided into three zones: EP, MP, and LP (see *Methods* for details). Overall, unc-18::AID* worms are able to both induce and repair DBSs at similar frequencies to wild type. The transition zone did display a slight, but significant, decrease in RAD-51 foci in unc-18::AID* worms (average RAD-51 foci: 0.9 ± 1.7 SD) compared to wild type (average RAD-51 foci: 1.5 ± 2.3SD; *P* < 0.0001, Kruskal–Wallis/Dunn’s multiple comparisons). However, RAD-51 foci numbers in early, mid, and late pachytene were indistinguishable from wild type in unc-18::AID* worms (average RAD-51 foci in unc-18::AID* EP: 4.3 ± 3.2SD, MP: 1.8 ± 2.5SD, LP: 0.7 ± 1.4SD; in wild type EP: 3.6 ± 2.9SD, MP: 1.6 ± 1.8SD, LP: 0.5 ± 1.0SD; EP *P* = 0.1647, MP *P* > 0.9999, LP *P* = 0.4720, Kruskal–Wallis/Dunn’s multiple comparisons). Taken together, formation of DSBs and subsequent off-loading of RAD-51 is largely unaffected by depletion of unc-18::AID* by auxin.

To determine if UNC-18::AID* depletion had any effect on crossover formation, we assayed for the presence of crossover events by quantifying the number of DAPI staining DNA bodies at diakinesis. Since *C. elegans* have six pairs of homologous chromosomes which are connected by crossovers to form bivalents at the diakinesis stage of meiotic prophase I, greater than 6 DAPI staining DNA bodies in diakinesis nuclei indicates errors in meiotic prophase I (*e.g.* lack of crossovers or chromosome fragmentation from unrepaired DSBs) (Villeneuve 1994). Both wild type and unc-18::AID* worms had an average of 6 DAPI staining DNA bodies at diakinesis (*P* > 0.99, Mann–Whitney), thereby indicating depletion of UNC-18::AID* has minimal effects on meiotic recombination. Taken together, subtle differences in both germline progression and brood size in UNC-18::AID* depletion do not have obvious negative consequences on meiosis.

### Conditional immobilization of hermaphrodites for live imaging

To immobilize worms for live imaging, 5–6 adult hermaphrodites carrying a fluorescent reporter and unc-18::AID*; rgef-1p::TIR1 were selected from 1 mM auxin plates. These worms were placed into a 1–2 µL drop of imaging M9 media containing 25 mM serotonin and 1 mM auxin on a poly-lysine coated coverslip. Previous studies have shown that serotonin is important to maintain motion within the germline during live imaging (Rog and Dernburg 2013; Rog and Dernburg 2015; Pattabiraman *et al.* 2017). Further, the addition of auxin within these media ensures a continued depletion of UNC-18::AID* throughout the imaging. Using M9 media, we made 7–9% agarose pads and this pad was then transferred and carefully laid over the top of the worm. Whatman paper was used to wick away any excess liquid between the coverslip and agarose pad. Then, the coverslip-worm-agar pad sandwich was sealed to a slide using Vaseline to prevent drying out of the agarose pad during live imaging (Supplementary Figure S1).

**Figure 4:**
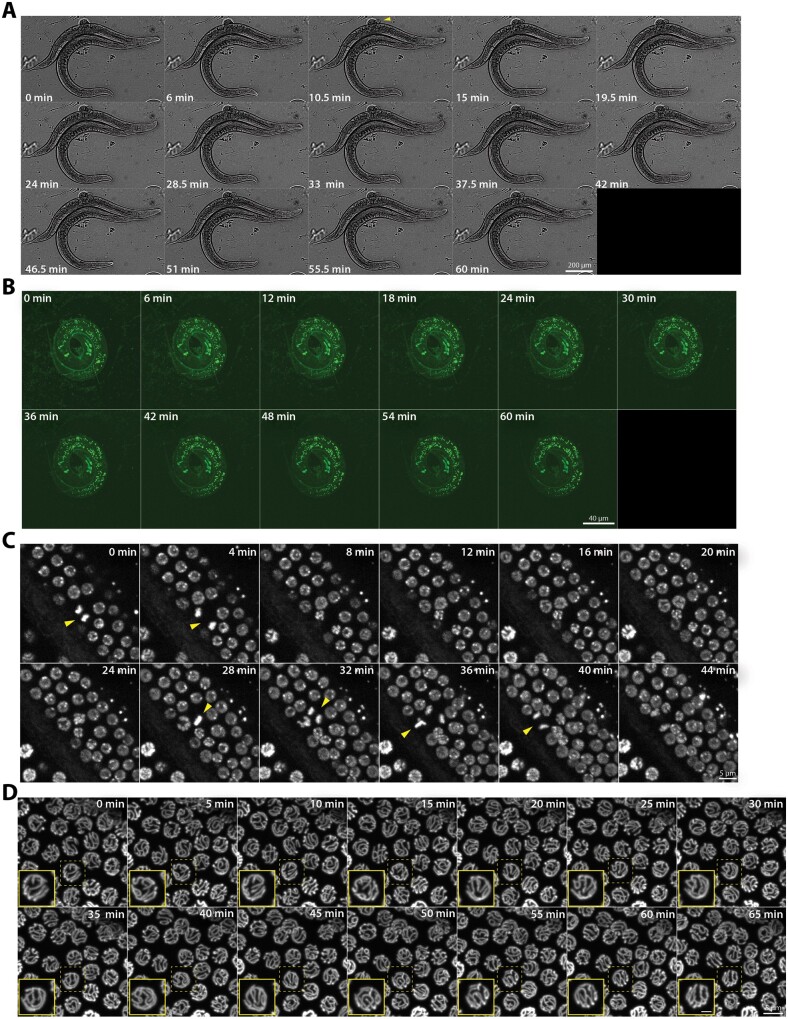
Conditional immobilization of hermaphrodites for live imaging. (A) Brightfield timelapse montage of an immobilized hermaphrodite worm at 40X magnification with images captured every 90 s for 60 min. The montage displays every third frame of the timelapse. Yellow arrowhead indicates ovulation of an egg by the immobilized hermaphrodite. (B) Autofluorescence from an immobilized L1 worm at 60X magnification with images captured every 2 min for 60 min. The montage displays every third frame of the timelapse. (C) mCherry::H2B in the germline of an immobilized L4 worm captured at 60X magnification with images captured every 2 min for 60 min. The montage displays every second frame of the timelapse for 44 min. Yellow arrowheads identify nuclei in the frame undergoing mitotic divisions. (D) SYP-2::GFP timelapse montage of the hermaphrodite germline at 60X magnification with images captured every 5 min for 65 min. The mid-pachytene region of the germline is shown and germline is moving from left to right in each image. The yellow-dashed box indicates the nucleus that is enlarged in the inset panel (yellow outline) with the scale bar in the inset panel representing 2 µm. The complete movies can be viewed in Supplementary Movies S1–S5.

For brightfield imaging, worms were imaged every 90 s for a total of 60 min. During that time, mounted hermaphrodite worms are able to subtly move their heads and some of the worms continued to ovulate embryos that would stack up in a pile next to the worm (2–3 oocytes/60 min, 7 worms, Figure 4A, Supplementary Movie S1). This continued ovulation of the hermaphrodite is an excellent indicator that germline progression is not being impeded by having the worms mounted underneath an agar pad.

Additionally, the same protocol can be used to immobilize and image larval worms. To minimize motion of the larval worms at 60X, we included 0.08% tricaine and 0.008% tetramisole anesthetics, which is at a concentration nearly 10-fold lower than previous studies (Wynne *et al.* 2012; Rog and Dernburg 2015; Pattabiraman *et al.* 2017; Rog *et al.* 2017). This addition of anesthetics allowed us to remove most, if not all, of the L1 larval worm motion when imaging (Figure 4B, Supplementary Movie S2). Further, we found that including this very small amount of anesthetics with knockdown of UNC-18::AID* in adult worms removed all of the residual head motion observed in the brightfield timelapse without anesthetics (compare Supplementary Movie S1 to Supplementary Movies S2, S4, and S5). Moreover, combining the low concentration anesthetics with UNC-18::AID* knockdown did not interfere with the postimaging recovery of either larval or adult worms.

The germline motion of both larval and adult animals appears to progress without impediment when using the unc-18::AID* immobilization system with anesthetics. Following immobilization, L4 larval germlines exhibited mitotic cellular divisions, which were visualized using a germline-specific mCherry::H2B transgene (Figure 4C, Supplementary Movie S3). Further, the movement of the adult germline can be seen by directly looking at the germline nuclei using a fluorescently tagged component of the synaptonemal complex (SC), SYP-2::GFP, which is a meiotic chromosome structure that assembles between homologous chromosomes in the germline from late transition zone to diakinesis (Figure 4D, Supplementary Movie S5) (Colaiacovo *et al.* 2003; Gao *et al.* 2016). Using SYP-2::GFP, we observed the previously described motion of the SC within each germ cell nucleus of living worms as well as the proximal movement of the nuclei away from the distal tip cell (Supplementary Movies S4 and S5) (Wynne *et al.* 2012; Rog and Dernburg 2013; Rog and Dernburg 2015; Pattabiraman *et al.* 2017). Notably, we can also observe the progressive motion of the diakinesis oocytes, which further indicates that oocyte progression is unimpeded by this immobilization method (Supplementary Movie S5).

### unc-18::AID* reversibly immobilizes male worms for live imaging

**Figure 5:**
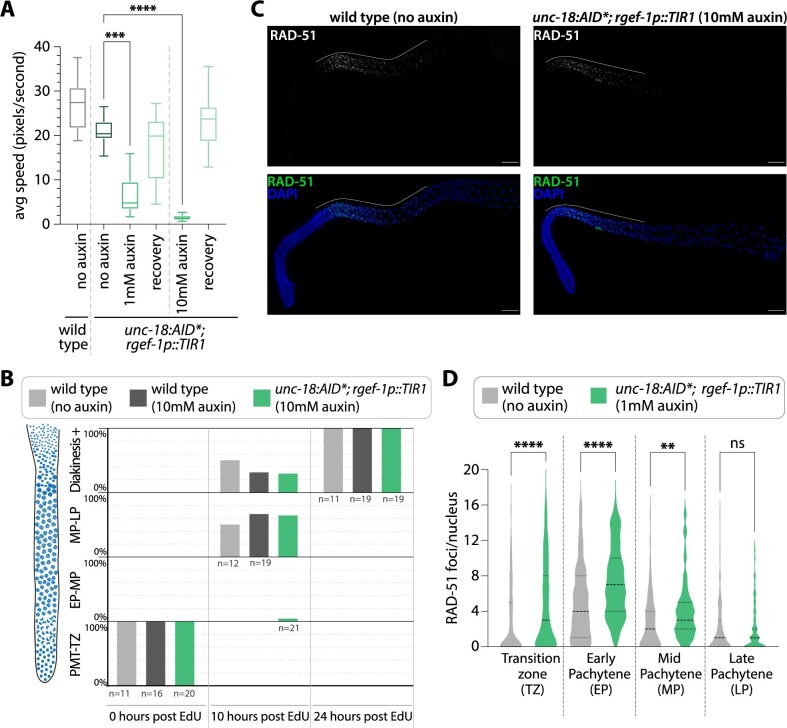
Conditional immobilization of males following auxin-dependent depletion of *unc-18.* (A) Quantification of the average speed in pixels per second of wild type (*n* = 24) and unc-18::AID*; rgef-1p::TIR1 on plates containing no auxin (*n* = 29), 1 mM auxin (*n* = 25), recovery from 1 mM auxin (*n* = 21), 10 mM auxin (*n* = 30), and recovery from 10 mM auxin (*n* = 21). The recovery category indicates worms that have been off auxin for 18–24 h prior to tracking worm motion. *** indicates *P* < 0.0001 and **** indicates *P* < 0.00001, Kruskal–Wallis/Dunn’s multiple comparisons. (B) Quantification of the nuclear progression through the germline in wild type with no auxin exposure and on 10-mM auxin plates and unc-18::AID*; rgef-1p::TIR1 on 10-mM auxin plates. Each gonad was scored based on the germline position of the last EdU stained nucleus from gonads dissected at 0, 10, and 24 h post-EdU labeling. For this analysis, the germline (diagrammed on the left) was divided into four regions: premeiotic tip to transition zone (PMT-TZ), early pachytene to mid pachytene (EP-MP), mid pachytene to late pachytene (MP-LP), and diakinesis to end of the germline (Diakinesis+). *N*-values for the number of worms scored are displayed on the plot below each bar. (C) Representative images of dissected gonads from wild type (no auxin exposure) and unc-18::AID*; rgef-1p::TIR1 (1 mM auxin exposure) males stained for RAD-51 (green) and DNA (DAPI, blue). The scale bar represents 20 µm and white line indicates the length of the RAD-51 staining within the germline. (D) Quantification of the number of RAD-51 foci per nucleus for wild type (grey, no auxin exposure, *n* = 6 gonads, 206 TZ nuclei, 151 EP nuclei, 100 MP nuclei, and 127 LP nuclei) and unc-18::AID*; rgef-1p::TIR1 (green, 10 mM auxin exposure, *n* = 6 gonads, 221 TZ nuclei, 115 EP nuclei, 94 MP nuclei, and 143 LP nuclei). Statistics determined using Kruskal–Wallis/Dunn’s multiple comparisons with * representing the number of significant digits after the decimal and “ns” meaning not significant.

One of the unique features of our conditional immobilization system using unc-18::AID*; rgef-1p::TIR1 is that it works well with whole, intact male worms. From our immobilization experiments, we found that male worms require a higher concentration of auxin to induce a more robust immobilization than hermaphrodite worms (Figure 5A). Male worms grown on plates containing 10 mM auxin exhibited a more severe immobilization phenotype and a tighter distribution of average speeds than male worms on 1 mM auxin (median average speed 1.251 and 4.809 pixels/s, respectively). Further, male worms removed from auxin for 18–24 h recovered sinusoidal movements close to wild type levels regardless of the initial auxin concentration. These results suggest a sexual dimorphic difference in auxin sensitivity in *C. elegans*, where male worms may absorb and process auxin differently than hermaphrodite worms. Although, recent study found that depletion of a different AID*-tagged protein (NHR-23) in the germline was equivalent between males and hermaphrodites on 4-mM auxin plates (Ragle *et al.* 2020). Thus, future studies focused on auxin processing in both *C. elegans* sexes may reveal the mechanisms behind this intriguing sexual dimorphism that may vary depending on the tissue assayed and AID*-tagged protein. Based on these analyses which revealed a more robust immobilization at higher auxin concentrations, we proceeded with using 10 mM auxin to immobilize male worms for live imaging.

Similar to hermaphrodites, we wanted to ensure that depleting UNC-18::AID* with the neuron expressed *TIR1* (*rgef-1p::TIR1*) did not cause detrimental changes to the *C. elegans* germline. To determine if depletion of UNC-18::AID* altered the progression of nuclei in the male germline, we used EdU labeling to track germ cell nuclei progression. Previous studies have demonstrated that the male germline progresses much faster than the hermaphrodite germline (24 h to complete spermatogenesis *vs* 48–72 h to complete oogenesis) (Jaramillo-Lambert *et al.* 2007). To monitor EdU-labeled nuclei progression within the germline, we dissected the germlines out of male worms following EdU soaking at 0, 10, and 24 h. Wild type male worms both in the presence and absence of 10 mM auxin displayed similar rates of germline progression, suggesting that auxin does not interfere with germline progression in males (Figure 5B). In addition, depletion of UNC-18::AID* did not appear to cause gross changes in male germline progression. Thus, overall progression of the male germline is relatively unaltered by the presence of both 10 mM auxin and depletion of UNC-18::AID*.

In addition to normal germline progression, the initiation and repair of DSBs exhibited a similar pattern to wild type following depletion of UNC-18 in the male germline. However, analysis of RAD-51 foci using immunofluorescence revealed that knockdown of UNC-18::AID* caused a slight increase in RAD-51 foci during both the transition zone and the beginning of pachytene (Figure 5C). In the transition zone, unc-18::AID* worms had on average about 2–3 more RAD-51 foci per nucleus than wild type (unc-18::AID* average RAD-51 foci: 4.5 ± 4.7SD, wild type: 2.5 ± 3.7SD; *P* < 0.0001, Kruskal–Wallis/Dunn’s multiple comparisons). This slight increase of approximately 2–3 RAD-51 foci in unc-18::AID* worms persisted through early pachytene and mid pachytene, but by late pachytene, both unc-18::AID* worms and wild type displayed similar amounts of RAD-51 foci (average RAD-51 foci in unc-18::AID* EP: 7.3 ± 3.9SD, MP: 4.0 ± 3.3SD, LP: 1.9 ± 2.6SD; in wild type EP: 4.8 ± 4.0SD, MP: 2.6 ± 2.9SD, LP: 1.8 ± 2.6SD; EP *P* < 0.0001, MP *P* = 0.0075, LP *P* > 0.9999, Kruskal–Wallis/Dunn’s multiple comparisons). Thus, the slight increase in DSBs in unc-18::AID* worms does not appear to affect the ability for these DNA breaks to be repaired by off-loading RAD-51 in late pachytene. Further, once removed from auxin, these unc-18::AID* males are able to mate and generate progeny (16 out of 20 males mated and 2 out of 20 males were unscorable since they went missing from the plates following 24 h off auxin). Taken together, the increase in DNA breaks following depletion of UNC-18::AID* does not appear to cause any large defects to spermatogenesis.

**Figure 6:**
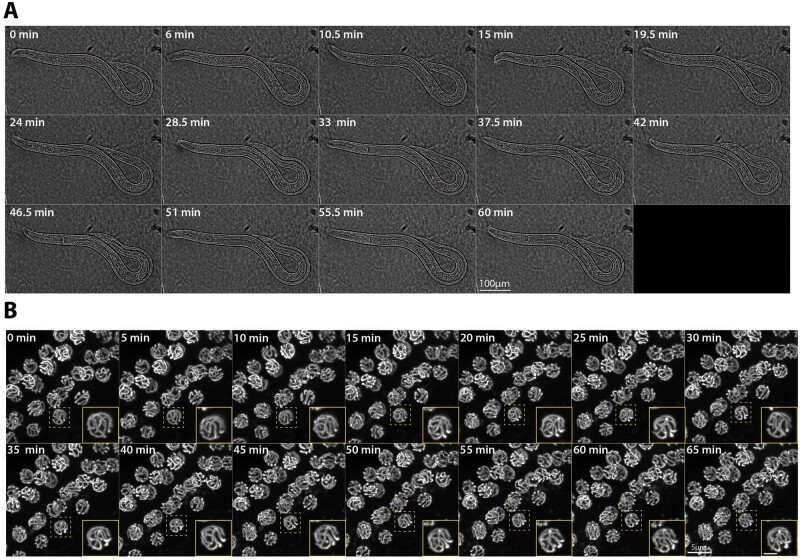
Conditional immobilization of males for live imaging. (A) Brightfield timelapse montage of an immobilized male worm at 20X magnification with images captured every 90 s for 60 min. The montage displays every third frame of the timelapse. (B) SYP-2::GFP timelapse montage of the male germline at 60X magnification with images captured every 5 min for 65 min. The mid-pachytene region of the germline is shown and germline is moving from bottom left to top right in each image. The yellow-dashed box indicates the nucleus that is enlarged in the inset panel (yellow outline) with the scale bar in the inset panel representing 2 µm. The complete movies can be viewed in Supplementary Movies S6–S8.

To enable live imaging of male worms, we mounted the worms using the same steps as described above for the hermaphrodite worms except we used 10 mM auxin in the M9 media instead of 1 mM auxin to maintain depletion of UNC-18::AID* during imaging (Supplementary Figure S1). All live imaging experiments were performed using the same imaging settings as the hermaphrodites except for the brightfield timelapses, which due to the smaller size of the male worms were captured at 20X magnification instead of 10X (Figure 6). Notably, the same mounting and immobilization method worked as efficiently to immobilize the male worms as it did with the hermaphrodite worms (Figure 6A, Supplementary Movie S6). Further, using SYP-2::GFP with the unc-18::AID*; rgef-1::TIR1 conditional immobilization technique, we were able to observe both the directional motion of germline progression and the chromosome motion within each nucleus (Figure 6B, Supplementary Movies S7 and S8).

## Discussion

The transparent nature of the *C. elegans* worm makes this model organism ideal for live imaging studies, however, effectively and reliably immobilizing the worm without injury has been a challenge for many *C. elegans* labs seeking to do live imaging experiments. We developed and validated a new tool that enables conditional immobilization of *C. elegans* for live imaging. This conditional immobilization tool uses the AID system, which we show works for immobilizing both larval and adult hermaphrodite and male worms. Notably, we found that depletion of the gene product responsible for this immobilization phenotype does not cause any substantial changes within the germline of either sex. Finally, with this tool, we were able to demonstrate that both male and hermaphrodite adult worms can be minimally restrained as whole animals with an agar pad and imaged live for at least 2 h (Supplementary Movies S5 and S8).

The conditional immobilization technique described here to immobilize worms enhances the existing toolkit for live imaging worms. While many modalities exist from microfluidic chips to pharmaceuticals for immobilization of the worm, here, we present an accessible genetic tool that can be used to easily immobilize worms and can be implemented in any lab without needing to purchase specialized equipment or use hazardous chemicals. Notably, the use of anesthetics has been widespread for live imaging studies; however, male *C. elegans* are known to respond differently than hermaphrodites to different chemicals and toxins including the widely used anesthetic levamisole (Lopes *et al.* 2008; Ruszkiewicz *et al.* 2019). Thus, methods used to immobilize hermaphrodite worms may not be as effective against male worms and have inhibited live imaging of sexual dimorphisms and male-specific processes. A recent study demonstrated that male worms can be immobilized for live imaging of the spermatocyte divisions using an agar pad approach (Fabig *et al.* 2020). After incorporating a modified version of this agar pad approach with our conditional immobilization tool (see *Methods*), we were able to consistently and reliably mount male worms without any of the technical challenges associated with the agar pad handling or placement. Specifically with our combination of these techniques, we found that regardless of how the agar pad was placed over the males, all of the mounted male worms were suitable for imaging and remained immobile. Further, our auxin-based immobilization system could be combined with other standardized mounting procedures. Overall, multiple options for immobilization of male worms increase the possibility of live imaging experiments that can be performed in a multitude of laboratory settings with different resources.

Our conditional immobilization tool works to efficiently immobilize both males and hermaphrodites for live imaging worms for sex-specific comparison experiments. For the *C. elegans* germline, an increasing number of studies are indicating hermaphrodites and males have multiple sexually dimorphic features that lead to differential regulation of genome integrity germline (Chu *et al.* 2006; Stanfield and Villeneuve 2006; Jaramillo-Lambert *et al.* 2007; Shakes *et al.* 2009; Jaramillo-Lambert *et al.* 2010; Checchi *et al.* 2014; Jaramillo-Lambert *et al.* 2016; Cahoon and Libuda 2019; Kurhanewicz *et al.* 2020; Li *et al.* 2020). To our knowledge, the live images of the SC in males may represent the first time the SC has been observed within living *C. elegans* spermatocytes. This conditional immobilization tool enables future experiments examining the dynamics of the SC between the sexes and has the potential to uncover new sexual dimorphic features of the *C. elegans* germline. Further, this method can be used to examine additional sexual dimorphisms in the germline such as germline progression, chromosome compaction, meiotic cell divisions, and the endogenous RNAi system. Moreover, sexual dimorphisms occur outside of the germline and this system can be also used to live image the development of other tissues within the worm such as the digestive tract and muscles, both of which display sexually dimorphic features during development and within the adult animal (Emmons 2014).

Our immobilization system has both flexibility for use in a variety of experiments and the ability for future modifications and expansions. To enable live imaging of any existing tagged fluorescence reporter strains, the components of our live imaging system can easily be incorporated through either genetic crosses or CRISPR-Cas9 injections (see *Methods* and Supplementary Table S1 for CRISPR details). Further, genetically linking the *rgef-1p::TIR1* transgene to the unc-18:AID* locus will simplify moving this system into existing tagged lines through genetic crosses. Moreover, there are likely more genes within the *C. elegans* genome that could be tagged with AID and used to induce immobilization analogous to UNC-18 depletion*.* Identification of additional genes that cause paralysis on different chromosomes and in different tissues will permit further flexibility with this immobilization system. Currently, this immobilization system works well for visualization of the germline but may not be ideal for neuronal studies since UNC-18 is important for nervous system function. However, many genes within the worm have been identified to cause severe paralysis and some of these do not alter the nervous system, such as *unc-54* which is part of the muscle myosin complex. Thus, future expansion of this conditional immobilization system by AID tagging other genes will provide even greater genetic flexibility for use of this system in a multitude of live imaging studies.

## Data availability

All strains are available upon request. Strains developed as part of this study (*i.e.*, DLW104, DLW110, DLW114, and DLW124) are available at the CGC. All Supplemental materials are available at figshare: https://doi.org/10.25387/g3.14673441. Supplementary Figure S1 shows a diagram of the steps used to immobilize worms for live imaging. Supplementary Table S1 contains all the primers and sgRNAs used for CRIPSR generations of unc-18::AID*, unc-104::AID*, and AID*::unc-52. Supplementary Movie S1 is a brightfield timelapse of an immobilized hermaphrodite at 10X magnification. Supplementary Movie S2 is a timelapse of the autofluorescence from an immobilized L1 worm. Supplementary Movie S3 is a timelapse of mCherry::H2B in the germline of an L4 worm. Supplementary Movie S4 is a timelapse of SYP-2::GFP in an immobilized hermaphrodite at 60× magnification. Supplementary Movie S5 is an entire germline view of SYP-2::GFP at 60Xin an immobilized hermaphrodite. Supplementary Movie S6 is a brightfield timelapse of an immobilized male at 20X magnification. Supplementary Movie S7 is a timelapse of SYP-2::GFP in an immobilized male at 60X magnification. Supplementary Movie S8 is an entire germline view of SYP-2::GFP at 60X in an immobilized male.

## References

[jkab310-B1] Almanzar DE , Gordon SG, Rog O. 2021. Meiotic sister chromatid exchanges are rare in *C. elegans*. Curr Biol. 31:1499–1507.e3. 33740426 10.1016/j.cub.2020.11.018 PMC8051885

[jkab310-B2] Ashley GE , Duong T, Levenson MT, Martinez MAQ, Johnson LC, et al 2021. An expanded auxin-inducible degron toolkit for *Caenorhabditis elegans*. Genetics. 217:iyab006. 10.1093/genetics/iyab006 PMC8045686 33677541

[jkab310-B3] Avery L , Shtonda BB. 2003. Food transport in the *C. elegans* pharynx. J Exp Biol. 206:2441–2457. 12796460 10.1242/jeb.00433 PMC3951750

[jkab310-B4] Barr MM , García LR, Portman DS. 2018. Sexual dimorphism and sex differences in *Caenorhabditis elegans* neuronal development and behavior. Genetics. 208:909–935. 29487147 10.1534/genetics.117.300294 PMC5844341

[jkab310-B5] Burnett K , Edsinger E, Albrecht DR. 2018. Rapid and gentle hydrogel encapsulation of living organisms enables long-term microscopy over multiple hours. Commun Biol. 1:73. 30271954 10.1038/s42003-018-0079-6 PMC6123791

[jkab310-B6] Cahoon CK , Libuda DE. 2019. Leagues of their own: sexually dimorphic features of meiotic prophase I. *Chromosoma*. 128:199–214. 10.1007/s00412-019-00692-x PMC6823309 30826870

[jkab310-B7] Chalfie M. 2009. GFP: lighting up life. Proc Natl Acad Sci U S A. 106:10073–10080. 19553219 10.1073/pnas.0904061106 PMC2700921

[jkab310-B8] Checchi PM , Lawrence KS, Van MV, Larson BJ, Engebrecht J. 2014. Pseudosynapsis and decreased stringency of meiotic repair pathway choice on the hemizygous sex chromosome of *Caenorhabditis elegans* males. Genetics. 197:543–560. 24939994 10.1534/genetics.114.164152 PMC4063914

[jkab310-B9] Chu DS , Liu H, Nix P, Wu TF, Ralston EJ, et al 2006. Sperm chromatin proteomics identifies evolutionarily conserved fertility factors. Nature. 443:101–105. 16943775 10.1038/nature05050 PMC2731558

[jkab310-B10] Chung K , Crane MM, Lu H. 2008. Automated on-chip rapid microscopy, phenotyping and sorting of *C. elegans*. Nat Methods. 5:637–643. 18568029 10.1038/nmeth.1227

[jkab310-B11] Colaiacovo MP , MacQueen AJ, Martinez-Perez E, McDonald K, Adamo A, et al 2003. Synaptonemal complex assembly in *C. elegans* is dispensable for loading strand-exchange proteins but critical for proper completion of recombination. Dev Cell. 5:463–474. 12967565 10.1016/s1534-5807(03)00232-6

[jkab310-B12] Crittenden SL , Leonhard KA, Byrd DT, Kimble J. 2006. Cellular analyses of the mitotic region in the *Caenorhabditis elegans* adult germ line. Mol Biol Cell. 17:3051–3061. 16672375 10.1091/mbc.E06-03-0170 PMC1552046

[jkab310-B13] Dong L , Cornaglia M, Krishnamani G, Zhang J, Mouchiroud L, et al 2018. Reversible and long-term immobilization in a hydrogel-microbead matrix for high-resolution imaging of *Caenorhabditis elegans* and other small organisms. PLoS One. 13:e0193989. 29509812 10.1371/journal.pone.0193989 PMC5839568

[jkab310-B14] Emmons SW. 2014. The development of sexual dimorphism: studies of the *Caenorhabditis elegans* male. Wiley Interdiscip Rev Dev Biol. 3:239–262. 25262817 10.1002/wdev.136 PMC4181595

[jkab310-B15] Fabig G , Löffler F, Götze C, Müller-Reichert T. 2020. Live-cell imaging and quantitative analysis of meiotic divisions in *Caenorhabditis elegans* males. Bio Protoc. 10:e3785. 10.21769/BioProtoc.3785 PMC7842533 33659440

[jkab310-B16] Fox PM , Vought VE, Hanazawa M, Lee MH, Maine EM, et al 2011. Cyclin E and CDK-2 regulate proliferative cell fate and cell cycle progression in the *C. elegans* germline. Development. 138:2223–2234. 21558371 10.1242/dev.059535 PMC3091494

[jkab310-B17] Gao J , Barroso C, Zhang P, Kim HM, Li S, et al 2016. N-terminal acetylation promotes synaptonemal complex assembly in *C. elegans*. Genes Dev. 30:2404–2416. 27881602 10.1101/gad.277350.116 PMC5131780

[jkab310-B18] Han X , Su Y, White H, O'Neill KM, Morgan NY, et al 2021. A polymer index-matched to water enables diverse applications in fluorescence microscopy. Lab Chip. 21:1549–1562. 33629685 10.1039/d0lc01233e PMC8058278

[jkab310-B19] Hayashi K , Matsumoto S, Miyamoto MG, Niwa S. 2019. Physical parameters describing neuronal cargo transport by kinesin UNC-104. Biophys Rev. 11:471–482. 31115864 10.1007/s12551-019-00548-9 PMC6558098

[jkab310-B20] Hillers KJ , Jantsch V, Martinez-Perez E, Yanowitz JL. 2017. Meiosis. WormBook. 2017;Doi: 10.1895/wormbook.1.178.1. 10.1895/wormbook.1.178.1 PMC5215044 26694509

[jkab310-B21] Hotzi B , Kosztelnik M, Hargitai B, Takács-Vellai K, Barna J, et al 2018. Sex-specific regulation of aging in *Caenorhabditis elegans*. Aging Cell. 17:e12724. 29493066 10.1111/acel.12724 PMC5946081

[jkab310-B22] Hunter N. 2015. Meiotic recombination: the essence of heredity. Cold Spring Harb Perspect Biol.7:a016618. 10.1101/cshperspect.a016618 PMC4665078 26511629

[jkab310-B23] Jaramillo-Lambert A , Ellefson M, Villeneuve AM, Engebrecht J. 2007. Differential timing of S phases, *X* chromosome replication, and meiotic prophase in the *C. elegans* germ line. Dev Biol. 308:206–221. 17599823 10.1016/j.ydbio.2007.05.019

[jkab310-B24] Jaramillo-Lambert A , Fabritius AS, Hansen TJ, Smith HE, Golden A. 2016. The identification of a novel mutant allele of topoisomerase II in *Caenorhabditis elegans* reveals a unique role in chromosome segregation during spermatogenesis. Genetics. 204:1407–1422. 27707787 10.1534/genetics.116.195099 PMC5161275

[jkab310-B25] Jaramillo-Lambert A , Harigaya Y, Vitt J, Villeneuve A, Engebrecht J. 2010. Meiotic errors activate checkpoints that improve gamete quality without triggering apoptosis in male germ cells. Curr Biol. 20:2078–2089. 20970339 10.1016/j.cub.2010.10.008 PMC3005853

[jkab310-B26] Kasimatis KR , Moerdyk-Schauwecker MJ, Phillips PC. 2018. Auxin-mediated sterility induction system for longevity and mating studies in *Caenorhabditis elegans*. G3 (Bethesda). 8:2655–2662. 29880556 10.1534/g3.118.200278 PMC6071612

[jkab310-B27] Kurhanewicz NA , Dinwiddie D, Bush ZD, Libuda DE. 2020. Elevated temperatures cause transposon-associated DNA damage in *C. elegans* spermatocytes. Curr Biol. 30:5007–5017.e4. 33065011 10.1016/j.cub.2020.09.050 PMC9191688

[jkab310-B28] Li Q , Hariri S, Engebrecht J. 2020. Meiotic double-strand break processing and crossover patterning are regulated in a sex-specific manner by BRCA1-BARD1 in *Caenorhabditis elegans*. Genetics. 216:359–379. 32796008 10.1534/genetics.120.303292 PMC7536853

[jkab310-B29] Libuda DE , Uzawa S, Meyer BJ, Villeneuve AM. 2013. Meiotic chromosome structures constrain and respond to designation of crossover sites. Nature. 502:703–706. 24107990 10.1038/nature12577 PMC3920622

[jkab310-B30] Lipton J , Kleemann G, Ghosh R, Lints R, Emmons SW. 2004. Mate searching in *Caenorhabditis elegans*: a genetic model for sex drive in a simple invertebrate. J Neurosci. 24:7427–7434. 15329389 10.1523/JNEUROSCI.1746-04.2004 PMC6729642

[jkab310-B31] Lopes PC , Sucena E, Santos ME, Magalhães S. 2008. Rapid experimental evolution of pesticide resistance in *C. elegans* entails no costs and affects the mating system. PLoS One. 3:e3741. 19011681 10.1371/journal.pone.0003741 PMC2580027

[jkab310-B32] Martinez MAQ , Kinney BA, Medwig-Kinney TN, Ashley G, Ragle JM, et al 2020. Rapid degradation of *Caenorhabditis elegans* proteins at single-cell resolution with a synthetic auxin. G3 (Bethesda). 10:267–280. 31727633 10.1534/g3.119.400781 PMC6945041

[jkab310-B33] Morgan DE , Crittenden SL, Kimble J. 2010. The *C. elegans* adult male germline: stem cells and sexual dimorphism. Dev Biol. 346:204–214. 20659446 10.1016/j.ydbio.2010.07.022 PMC2945412

[jkab310-B34] Mullenders LHF. 2018. Solar UV damage to cellular DNA: from mechanisms to biological effects. Photochem Photobiol Sci. 17:1842–1852. 30065996 10.1039/c8pp00182k

[jkab310-B35] Muschiol D , Schroeder F, Traunspurger W. 2009. Life cycle and population growth rate of *Caenorhabditis elegans* studied by a new method. BMC Ecol. 9:14. 19445697 10.1186/1472-6785-9-14 PMC2696410

[jkab310-B36] Natsume T , Kanemaki MT. 2017. Conditional degrons for controlling protein expression at the protein level. Annu Rev Genet. 51:83–102. 29178817 10.1146/annurev-genet-120116-024656

[jkab310-B37] Nika L , Gibson T, Konkus R, Karp X. 2016. Fluorescent beads are a versatile tool for staging *Caenorhabditis elegans* in different life histories. G3 (Bethesda). 6:1923–1933. 27172224 10.1534/g3.116.030163 PMC4938646

[jkab310-B38] Nussbaum-Krammer CI , Neto MF, Brielmann RM, Pedersen JS, Morimoto RI. 2015. Investigating the spreading and toxicity of prion-like proteins using the metazoan model organism *C. elegans*. J Vis Exp. 95:52321. 25591151 10.3791/52321 PMC4354510

[jkab310-B39] Park S , Bin N-R, Yu B, Wong R, Sitarska E, et al 2017. UNC-18 and tomosyn antagonistically control synaptic vesicle priming downstream of UNC-13 in *Caenorhabditis elegans*. J Neurosci. 37:8797–8815. 28821673 10.1523/JNEUROSCI.0338-17.2017 PMC5588468

[jkab310-B40] Pattabiraman D , Roelens B, Woglar A, Villeneuve AM. 2017. Meiotic recombination modulates the structure and dynamics of the synaptonemal complex during *C. elegans* meiosis. PLoS Genet. 13:e1006670. 28339470 10.1371/journal.pgen.1006670 PMC5384771

[jkab310-B41] Pelisch F , Tammsalu T, Wang B, Jaffray EG, Gartner A, et al 2017. A SUMO-dependent protein network regulates chromosome congression during oocyte meiosis. Mol Cell. 65:66–77. 27939944 10.1016/j.molcel.2016.11.001 PMC5222697

[jkab310-B42] Preibisch S , Saalfeld S, Tomancak P. 2009. Globally optimal stitching of tiled 3D microscopic image acquisitions. Bioinformatics. 25:1463–1465. 19346324 10.1093/bioinformatics/btp184 PMC2682522

[jkab310-B43] Ragle JM , Aita AL, Morrison KN, Martinez-Mendez R, Saeger HN, et al 2020. The conserved molting/circadian rhythm regulator NHR-23/NR1F1 serves as an essential co-regulator of *C. elegans* spermatogenesis. Development. 147:dev193862. 10.1242/dev.193862 PMC7710015 33060131

[jkab310-B44] Reinke V , Smith HE, Nance J, Wang J, Van Doren C, et al 2000. A global profile of germline gene expression in *C. elegans*. Mol Cell. 6:605–616. 11030340 10.1016/s1097-2765(00)00059-9

[jkab310-B45] Remington SJ. 2011. Green fluorescent protein: a perspective. Protein Sci. 20:1509–1519. 21714025 10.1002/pro.684 PMC3190146

[jkab310-B46] Rivera Gomez KA , Schvarzstein M. 2018. Immobilization of nematodes for live imaging using an agarose pad produced with a Vinyl Record. microPublication Biology. Doi: 10.17912/QG0J-VT85. 10.17912/QG0J-VT85 PMC7282523 32550397

[jkab310-B47] Robinson JD , Powell JR. 2016. Long-term recovery from acute cold shock in *Caenorhabditis elegans*. BMC Cell Biol. 17:2. 26754108 10.1186/s12860-015-0079-z PMC4709947

[jkab310-B48] Rog O , Dernburg AF. 2013. Chromosome pairing and synapsis during *Caenorhabditis elegans* meiosis. Curr Opin Cell Biol. 25:349–356. 23578368 10.1016/j.ceb.2013.03.003 PMC3694717

[jkab310-B49] Rog O , Dernburg AF. 2015. Direct visualization reveals kinetics of meiotic chromosome synapsis. Cell Rep. 10:1639–1645. 25772351 10.1016/j.celrep.2015.02.032 PMC4565782

[jkab310-B50] Rog O , Kohler S, Dernburg AF. 2017. The synaptonemal complex has liquid crystalline properties and spatially regulates meiotic recombination factors. Elife. 6:e21455. 28045371 10.7554/eLife.21455 PMC5268736

[jkab310-B51] Rogalski TM , Mullen GP, Bush JA, Gilchrist EJ, Moerman DG. 2001. UNC-52/perlecan isoform diversity and function in *Caenorhabditis elegans*. Biochem Soc Trans. 29:171–176. 11356148 10.1042/0300-5127:0290171

[jkab310-B52] Rosu S , Libuda DE, Villeneuve AM. 2011. Robust crossover assurance and regulated interhomolog access maintain meiotic crossover number. Science. 334:1286–1289. 22144627 10.1126/science.1212424 PMC3360972

[jkab310-B53] Ruszkiewicz JA , Teixeira de Macedo G, Miranda-Vizuete A, Bowman AB, Bornhorst J, et al 2019. Sex-specific response of *Caenorhabditis elegans* to methylmercury toxicity. Neurotox Res. 35:208–216. 30155682 10.1007/s12640-018-9949-4

[jkab310-B54] San-Miguel A , Lu H. 2013. Microfluidics as a tool for *C. elegans* research.. WormBook. 2013;Doi: 10.1895/wormbook.1.162.1. 10.1895/wormbook.1.162.1 PMC4781173 24065448

[jkab310-B55] Schedl T , Kimble J. 1988. fog-2, a germ-line-specific sex determination gene required for hermaphrodite spermatogenesis in *Caenorhabditis elegans*. Genetics. 119:43–61. 3396865 10.1093/genetics/119.1.43 PMC1203344

[jkab310-B56] Schindelin J , Arganda-Carreras I, Frise E, Kaynig V, Longair M, et al 2012. Fiji: an open-source platform for biological-image analysis. Nat Methods. 9:676–682. 22743772 10.1038/nmeth.2019 PMC3855844

[jkab310-B57] Seidel HS , Kimble J. 2015. Cell-cycle quiescence maintains *Caenorhabditis elegans* germline stem cells independent of GLP-1/Notch. Elife. 4:e10832. 10.7554/eLife.10832 PMC4718729 26551561

[jkab310-B58] Serrano-Saiz E , Leyva-Díaz E, De La Cruz E, Hobert O. 2018. BRN3-type POU homeobox genes maintain the identity of mature postmitotic neurons in nematodes and mice. Curr Biol. 28:2813–2823.e2. 30146154 10.1016/j.cub.2018.06.045

[jkab310-B59] Shakes DC , Wu JC, Sadler PL, Laprade K, Moore LL, et al 2009. Spermatogenesis-specific features of the meiotic program in *Caenorhabditis elegans*. PLoS Genet. 5:e1000611. 19696886 10.1371/journal.pgen.1000611 PMC2720455

[jkab310-B60] Stanfield GM , Villeneuve AM. 2006. Regulation of sperm activation by SWM-1 is required for reproductive success of *C. elegans* males. Curr Biol. 16:252–263. 16461278 10.1016/j.cub.2005.12.041

[jkab310-B61] Toraason E , Adler VL, Kurhanewicz NA, DiNardo A, Saunders AM, et al 2021. Automated and customizable quantitative image analysis of whole *Caenorhabditis elegans* germlines. Genetics. 217:iyab010. 10.1093/genetics/iyab010 PMC8045727 33772283

[jkab310-B62] Villeneuve AM. 1994. A cis-acting locus that promotes crossing over between X chromosomes in *Caenorhabditis elegans*. Genetics. 136:887–902. 8005443 10.1093/genetics/136.3.887 PMC1205894

[jkab310-B63] Wang Y , Yu Z, Cahoon CK, Parmely T, Thomas N, et al 2018. Combined expansion microscopy with structured illumination microscopy for analyzing protein complexes. Nat Protoc. 13:1869–1895. 30072723 10.1038/s41596-018-0023-8

[jkab310-B64] Ward A , Liu J, Feng Z, Xu XZS. 2008. Light-sensitive neurons and channels mediate phototaxis in *C. elegans*. Nat Neurosci. 11:916–922. 18604203 10.1038/nn.2215 PMC2652401

[jkab310-B65] Ward S , Carrel JS. 1979. Fertilization and sperm competition in the nematode *Caenorhabditis elegans*. Dev Biol. 73:304–321. 499670 10.1016/0012-1606(79)90069-1

[jkab310-B66] Wynne DJ , Rog O, Carlton PM, Dernburg AF. 2012. Dynein-dependent processive chromosome motions promote homologous pairing in *C. elegans* meiosis. J Cell Biol. 196:47–64. 22232701 10.1083/jcb.201106022 PMC3255982

[jkab310-B67] Zhang L , Ward JD, Cheng Z, Dernburg AF. 2015. The auxin-inducible degradation (AID) system enables versatile conditional protein depletion in *C. elegans*. Development. 142:4374–4384. 26552885 10.1242/dev.129635 PMC4689222

